# Meta-analysis of fMRI studies related to mathematical creativity

**DOI:** 10.3389/fpsyg.2024.1400328

**Published:** 2025-01-23

**Authors:** Qingqing Li, Sungyeun Kim

**Affiliations:** ^1^Department of Psychology, Northwest Normal University, Lanzhou, Gansu, China; ^2^Graduate School of Education, Incheon National University, Incheon, Republic of Korea

**Keywords:** creativity, fMRI, mathematics, mathematical creativity, meta-analysis

## Abstract

This study presents a comprehensive meta-analysis of fMRI data to explore the neural correlates of mathematical creativity, a vital competence in mathematics education. Utilizing Activation Likelihood Estimation (ALE) and Meta-Analytic Connectivity Modeling (MACM) techniques, we analyzed studies published up to 2022 to identify brain regions activated during mathematical and creative tasks. The findings reveal significant activation in the left inferior frontal gyrus (IFG) and the left superior frontal gyrus (SFG) during both mathematical and creative tasks, emphasizing their roles in idea generation, working memory, and executive control. The MACM analysis further highlights the importance of the frontoparietal network, a key player in cognitive control, for mathematical creativity. This network’s involvement in attention, working memory, and goal orientation aligns with the demands of mathematical problem-solving. Our results offer valuable insights into the neural mechanisms underlying mathematical creativity, providing a foundation for developing targeted educational strategies to enhance this crucial competence in learners.

## 1 Introduction

Mathematics is a core subject in most countries’ educational systems, and one of the goals of any educational system should be fostering creative people. Creativity plays an important role in the development of individuals and societies and is recognized as one of the key skills necessary for learners in the 21st century, as well as being a core attribute in contemporary economic, social, and educational environments (Swanzy-Impraim et al., 2022). Creativity is also recognized as an essential skill that students must master in higher education due to its direct relationship to the development of content knowledge and skills (Egan et al., 2017; Livingston, 2010). Creativity is defined as an ability to interact with cognitive abilities, personal qualities, and environmental factors to produce novel and effective ways of solving problems.

Doing meaningful science has been considered a creative act, and the nature of mathematics provides a suitable platform for developing creativity. Hence, the importance of mathematical creativity is apparent and needs no further emphasis, even though the definitions of mathematical creativity are various depending on researchers. Although research on creativity has been a hot topic, unfortunately, there has been not only a dearth of research on mathematical creativity, but even the definition of its concept still lacks a common definition (Mann, 2006; Tubb et al., 2020). Mathematical creativity is often considered a key component of mathematical ability, which includes flexible thinking, providing valuable and novel solutions to mathematical problems, analyzing mathematical problems in a variety of ways, the ability to perceive mathematical relationships using complex thinking, and generating original and novel mathematical products through identification and selection (Kattou et al., 2013; Sadak et al., 2022).

Yet the focus on mathematical creativity in mathematics education still never stops, such as the assessment of students’ mathematical creativity, how to better develop students’ mathematical creativity, and the eye-tracking characteristics of mathematical creativity (Schindler and Lilienthal, 2020; Suherman and Vidákovich, 2022). With the development of educational neuroscience, more and more studies have begun to explore the neural regions and networks of the brain that are related to creativity in an attempt to come up with more scientific educational strategies to improve students’ creativity because traditional educational research methods are difficult to investigate the relationship of mathematics and creativity despite providing some useful information (Agnoli et al., 2018; Sun et al., 2016; Zhou, 2018). Moreover, their relationship has never been explored explicitly in cognitive neuroscience.

Cognitive neuroscience can not only examine behavioral changes but also study the correlation of these behavioral changes with brain structure and function. This research approach can provide ample evidence for the effectiveness of educational interventions (Zhou, 2018). It has been suggested that mathematical creativity is a combination of general domain creativity and domain-specific mathematical ability (Schoevers et al., 2020). Based on this, this study intends to further investigate which educational strategies can scientifically and effectively promote students’ mathematical creativity based on exploring the cognitive neural basis of creativity and mathematical competence through a cognitive neural meta-analytic approach. Specifically, the research questions are as follows:

1. Which brain regions are activated during doing mathematical tasks and which brain regions are activated during doing creativity tasks?
2. Which brain regions overlap and which brain regions are unique?
3. How is the composition of brain networks related to overall mathematical creativity?

## 2 Materials and methods

### 2.1 Literature and methods

Potential articles were identified by searching empirical papers published by Web of Science, PLOS, APA through 2022. The research for articles related to mathematics keywords such as “mathematics,” “arithmetic,” “problem solving,” “addition,” “subtraction,” “division,” “multiplication,” “mental math,” and “numeral” were combined with technical terms such as “neuroimaging,” “brain imaging,” “cerebral correlates,” “neural correlates,” “functional magnetic resonance imaging” and its abbreviation. The research for articles related to creativity keywords such as “creativity,” “divergent thinking,” “creative idea generation,” “insight,” “metaphor,” “improvisation,” “aha,” “original idea,” “remote associates,” “heuristic prototype,” “creative thinking” AND “neuroimaging,” “brain imaging,” “cerebral correlates” “functional magnetic resonance imaging” and its abbreviation. We complemented these results with PubMed and NeuroQuery.

**Figure 1:**
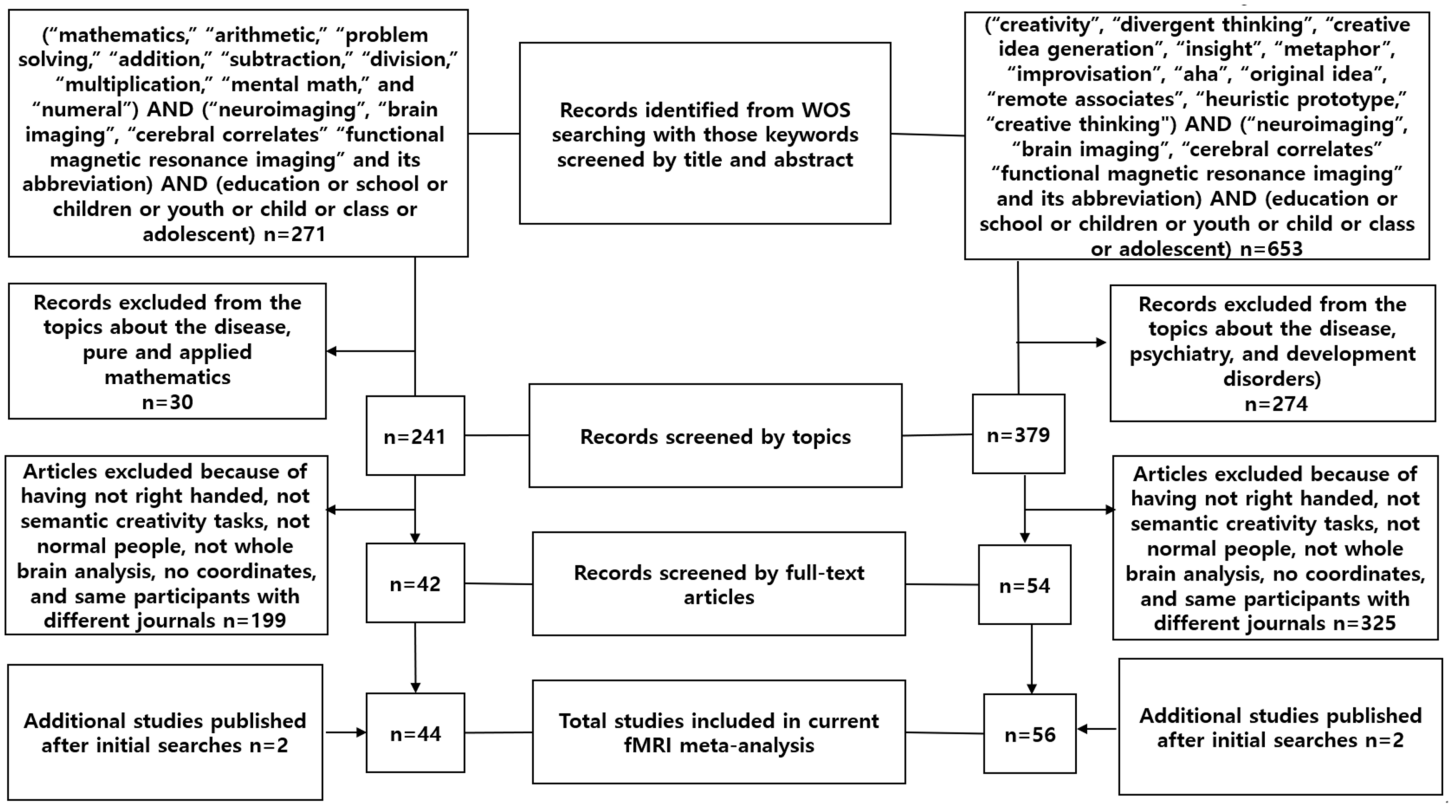
Brain regions activated during mathematical tasks or creative tasks.

Studies were eligible for the present meta-analysis included: (1) whole brain analysis had to be used and ROI studies were excluded, (2) MNI or Talairach coordinates had to be provided, (3) tasks were limited to mathematics and creativity, and (4) subjects had to be healthy and right-handed. A PRISMA flow chart of the selection process is illustrated below. A total of 44 papers on mathematics and 56 papers on creativity were included in the final meta-analysis (see Table 1; Figure 1).

**Table 1:** Summary of studies included in the meta-analysis.

1st Author (year)	Subjects	Male	Age	Mathematical task
Ansari (2006)	9	6	9.1–11.1	Nonsymbolic magnitude
Ashkenazi (2012)	17	6	7.0–9.0	Simple/complex addition
Ashkenazi (2022)	18	6	22.8	Estimation comparison
Ashkenazi (2022)	24	14	23.6	Estimation comparison
Baker (2015)	16	11	8.0–19.0	Relational calculation, reasoning
Caron (2020)	23	18	8.0–11.0	Binary/decimal learning
Chang (2018)	26	12	22.25	Arithmetic/nonarithmetic word
Cho (2011)	103	49	7.0–9.9	Univariate/multivariate addition
Davis (2009)	27	13	8.1	Exact calculation
Davis (2009)	24	12	8.2	Exact/approximate calculation
Declercq (2022)	26	13	10.4	Single/double digits
Dedovic (2009)	20	20	23	Experimental/control math
Du (2013)	39	19	10.1–11.3	Exact/approximate addition
Emerson (2012)	24	-	4.3–11.9	Formal/free matching
Grabner (2007)	25	25	25.38	Multiplication
Heidekum (2019)	46	17	23.6	Lexico-semantic/arithmetic interference
Holloway (2010)	19	7	6.8–9.3	Symbolic/nonsymbolic magnitude
Houde (2010)	88	-	8.1–12.5	Calculation
Houde (2011)	32	14	5.9–10.2	Number/color
Houde (2011)	16	6	5.2–7.2	Number/color
Isaacs (2001)	12	-	-	Calculation
Kawashima (2004)	8	4	9.0–14.0	Arithmetic
Kucian (2008)	20	10	9.2–12.0	Approximate/exact calculation
Li (2013)	34	16	9.6–11.3	Digits/beads/abacus
Libertus (2009)	15	8	8.0–9.0	Digit/letter and space
Matejko (2021)	38	21	7.7–10.4	Arithmetic/visuospatial working memory
McCaskey (2018)	11	6	9.1	Numerical order
Meintjes (2010)	16	6	8–12	Exact addition/proximity judgment
Michael (2010)	10	5	20	Linear/quadratic graph
Mondt (2011)	8	5	9.7	Simple/complex addition
Mussolin (2010)	15	9	10.9	Number/color comparison
Newman (2020)	43	22	7.5–9.1	Structured/free block play
Peters (2016)	23	12	9.0–12.0	Words/dots digits
Polspoel (2019)	20	15	9.79	Custom multiplication
Rosenberg-Lee (2011)	90	51	7.7–8.7	Arithmetic
Rosenberg-Lee (2018)	19	8	8.5	Arithmetic
Schwartz (2017)	17	10	8.2–13.7	Validity/likelihood trials
Skagenholt (2021)	44	20	23.69	Arabic/verbal/nonsymbolic magnitude
Soylu (2018)	24	12	8.4	Arithmetic
Vatansever (2020)	15	7	11.8	Number perception
Wakefield (2019)	20	8	7.0–9.0	Mathematical equivalence problems
Wang (2015)	29	15	7.4–8.6	Bead/picture match
Wilkey (2017)	36	22	18	Nonsymbolic number comparison
Zhou (2018)	24	11	21.5	Number/word/geometry
Abdul Hamid (2019)	50	-	-	Alternative use
Abraham (2012)	19	8	19–29	Alternative use
Abraham (2014)	28	14	22.8	Alternative use
Abraham (2014)	28	14	22.79	Alternative use
Abraham (2018)	43	-	-	Alternative use
Addis (2011)	15	8	18–33	Specific/general past/future events
Ahrens (2007)	8	8	21.0	Metaphor
Amir (2016)	22	19	20–47	Generate caption
Bambini (2011)	9	5	25	Metaphor
Beaty (2015)	25	12	18–30	Alternative use
Beaty (2017)	35	13	20.77	Metaphor
Benedek (2014)	35	11	18–29	Alternative use
Benedek (2016)	32	13	28.3	Sentence generation
Benedek (2018)	42	17	19–36	Alternative use
Berkowitz (2008)	12	-	21.9	Make up melodies/play patterns
Dandan (2013)	16	7	20–27	Related/unrelated prototypes
Darsaud (2011)	36	19	22.0	Number reduction
Edward (2021)	1	1(F)	12.0	Music improvisation
Ellamil (2012)	15	6	22.14	Designed book cover illustrations
Erhard (2014)	48	26	-	Brainstorming/creative writing
Eslinger (2009)	16	11	12.8	Cognitive activation
Fink (2010)	31	13	19–29	Alternative use
Fink (2012)	24	10	21–30	Alternative use
Frega (2013)	24	9	21.0	One word (listen, create, repeat)
Green (2009)	23	12	22.2	Analogy trials
Green (2012)	23	12	22.2	Analogical reasoning
Hahm (2017)	25	11	19.9	12 line drawing
Heinonen (2016)	16	4	19–49	Alternative use
Howard-Jones (2005)	8	1	19–28	Generate story
Huang (2013)	26	11	22	Visual imagination
Huang (2018)	20	9	21–26	Novelty/appropriateness of answer
Ivancovsky (2018)	36	-	28.93	Alternative use
Kaiser (2013)	19	7	21–34	Option generation
Kizilirmak (2016)	26	15	18–32	Alternative use
Kleibeuker (2017)	32	18	15–16	Alternative use
Kleibeuker (2017)	20	11	16.1	Matchstick problem
Kleibeuker (2017)	32	18	15–16	Alternative use
Kowatari (2009)	20	8	20–28	Designing new pens
Kuehn (2009)	25	-	17.3	Paired and single analogy
Luo (2003)	10	-	20–25	Semantic judgment
Luo (2003)	10	-	20–25	Music imaginary/performance
Madore (2019)	27	-	-	Alternative use
Mashal (2007)	15	8	21–31	Metaphor
Perchtold (2018)	45	14	18–34	Alternative use
Qiu (2010)	16	8	22.6	World riddle
Rapp (2004)	15	9	15.3	Metaphor
Rutter (2012)	18	9	22.78	Metaphor
Sakaki (2011)		10	21.71	Riddles
Shibata (2007)	13	8	21–29	Metaphor
Tian (2017)	24	13	18–25	Humorous/nonhumorous pictures
Villarreal (2013)	24	9	21.9	Create/repeat
Watson (2013)	23	9	18–27	Analogy
Wendelken (2008)	20	11	18–28	Analogical reasoning
Zhang (2014)	18	8	17–23	Generation of inventive conceptions
Zhao (2013)	18	7	23.6	‘chengyu’ riddle

### 2.2 ALE meta-analysis

As a coordinate-based meta-analysis ALE focuses on finding consistency in spatial location by counting the coordinate values reported in the article. The basic principle of ALE meta-analysis of coordinate information is to fit the activation points in each experiment to a probability distribution, that is, the magnitude of the probability that the activation points fall on each voxel of the brain (Eickhoff et al., 2009).

Significant activation of brain regions associated with mathematical creativity was detected using the ALE (Activation Likelihood Estimation) approach. A meta-analysis of mathematical tasks of a total of 958 foci reported in 44 experiments and creative tasks of a total of 1,177 foci reported in 56 was conducted using the ALE approach with GingerALE 3.0.2 software.^1^ Activation foci reported in Talairach space were converted to MNI space before being put into GingerALE. ALE-maps of single analyses were constructed using 1,000 permutations and a cluster level of FWE of *p* < 0.05 with a cluster forming a voxel level threshold of *p* < 0.001. ALE maps of contrast analyses were constructed using 5,000 permutations at *p* < 0.001 and a minimum volume of 50 mm^3^ (Müller et al., 2018). Each ALE map was visualized using the Mango 4.0.1 software.^2^

### 2.3 MACM analysis

Based on the brain regions derived from the findings of the ALE meta-analysis, and to explore the cognitive neural mechanisms of mathematical creativity, we performed a MACM analysis to detect synergistic activation patterns in these regions of interest. It allows for ALE analysis of BrainMap database (see Footnote 1) co-activation foci, which further reveals other important brain regions co-activated with the ROI through the functional connectivity of the task. MACM collects data from a variety of neural databases (e.g., Brain map) and utilizes meta-analysis algorithms, such as ALE, to assess or examine which brain regions are co-activated with a given seed region. The basic principle of MACM is that if two brain regions are functionally related, they are more likely to be activated by the same task (Kotkowski et al., 2018; Langner and Camilleri, 2021).

For the MACM analysis, we first selected the 9 regions derived from the ALE analysis described above as ROIs, including left precuneus, left inferior parietal lobule (IPL), right superior parietal lobule (SPL), left superior frontal gyrus (SFG), right insula, left insula, left precentral gyrus, left inferior frontal gyrus (IFG), left middle occipital gyrus (MOG), where the left inferior parietal lobule (IPL) and left superior frontal gyrus (SFG) is the region where creativity and mathematical ability are co-activated. Each ROI contains experiments, participants, and foci information as shown in Table 2. MACM map was visualized using the BrainNet Viewer software^3^ (Xia et al., 2013).

**Table 2:** Information from 9 ROIs selected from the Sleuth database.

Node	Region	Experiments	Participants	Foci
M1	Left Precuneus	25	416	355
M1:2C4	Left Inferior Parietal Lobule	109	1,504	1,544
M2	Right Superior Parietal Lobule	36	625	508
M3C2	Left Superior Frontal Gyrus	59	1,128	836
M4	Right Insula	91	1,243	1,123
M5	Left Insula	111	1,467	1,632
M6	Left Precentral Gyrus	86	1,109	1,240
C1	Left Inferior Frontal Gyrus	46	592	681
C3	Left Middle Occipital Gyrus	26	380	504

## 3 Results

### 3.1 Subsection

**Figure 2:**
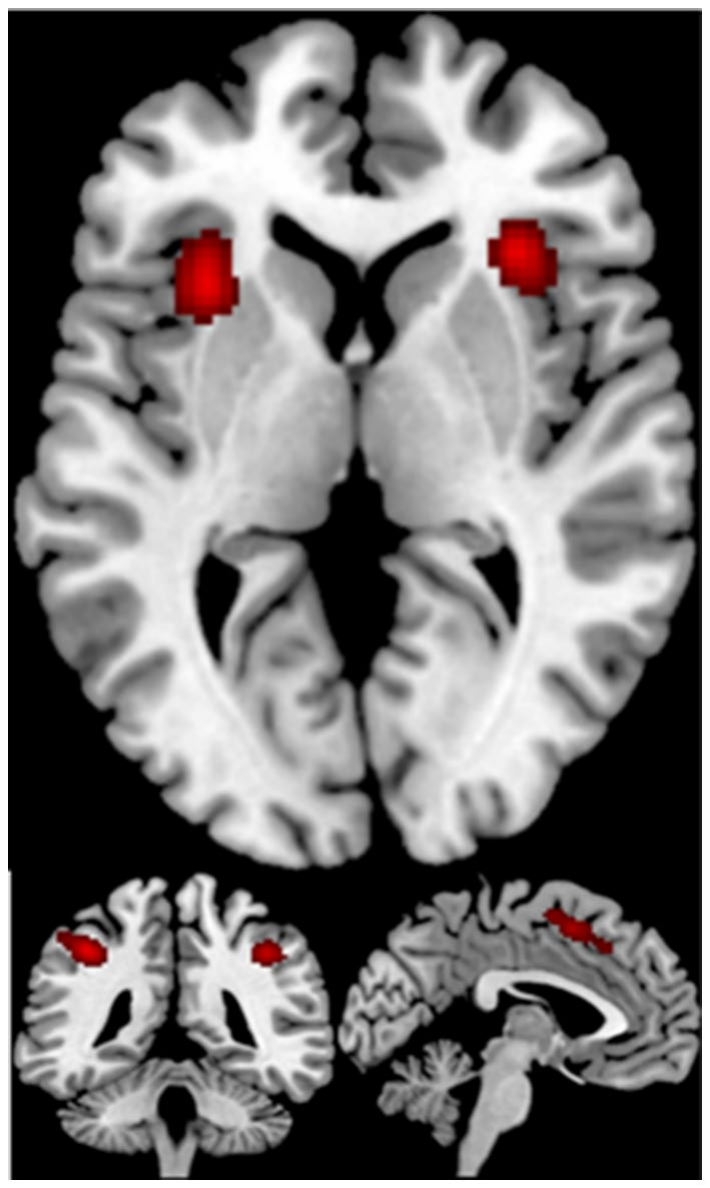
Displayed are significant results from the meta-analysis of mathematics.

The ALE meta-analysis on 44 studies for mathematics revealed 6 clusters of significant activation, including the bilateral inferior parietal lobule, the left superior parietal lobule, the left superior frontal gyrus, the right medial frontal gyrus, the left me-dial frontal gyrus, the bilateral insula, the left precentral gyrus (see Table 3; Figure 2).

**Table 3:** Regions of activations resulting from the meta-analysis of mathematics.

Cluster	Vol (mm^3^)	Region	H*	BA*	MNI coordinates			ALE
					x	y	z	
1	7,568	Precuneus	L	19	−30	−64	46	0.0513
		Inferior Parietal Lobule	L	40	−40	−42	46	0.0480
		Angular Gyrus	L	39	−32	−54	44	0.0407
2	5,808	Superior Parietal Lobule	R	7	32	−56	48	0.0455
		Inferior Parietal Lobule	R	40	40	−42	44	0.0416
		Precuneus	R	7	32	−60	36	0.0400
		Superior Parietal Lobule	R	7	32	−70	52	0.0254
3	5,096	Superior Frontal Gyrus	L	6	0	16	50	0.0369
		Medial Frontal Gyrus	R	8	4	26	42	0.0364
		Superior Frontal Gyrus	L	6	−2	12	50	0.0360
		Cingulate Gyrus	R	32	8	24	38	0.0336
		Medial Frontal Gyrus	L	6	−2	0	58	0.0283
4	3,440	Insula	R	13	34	24	2	0.0544
		Claustrum	R	*	32	20	12	0.0347
5	2,920	Insula	L	13	−32	18	6	0.0512
6	1728	Precentral Gyrus	L	6	−44	2	32	0.0395

**Figure 3:**
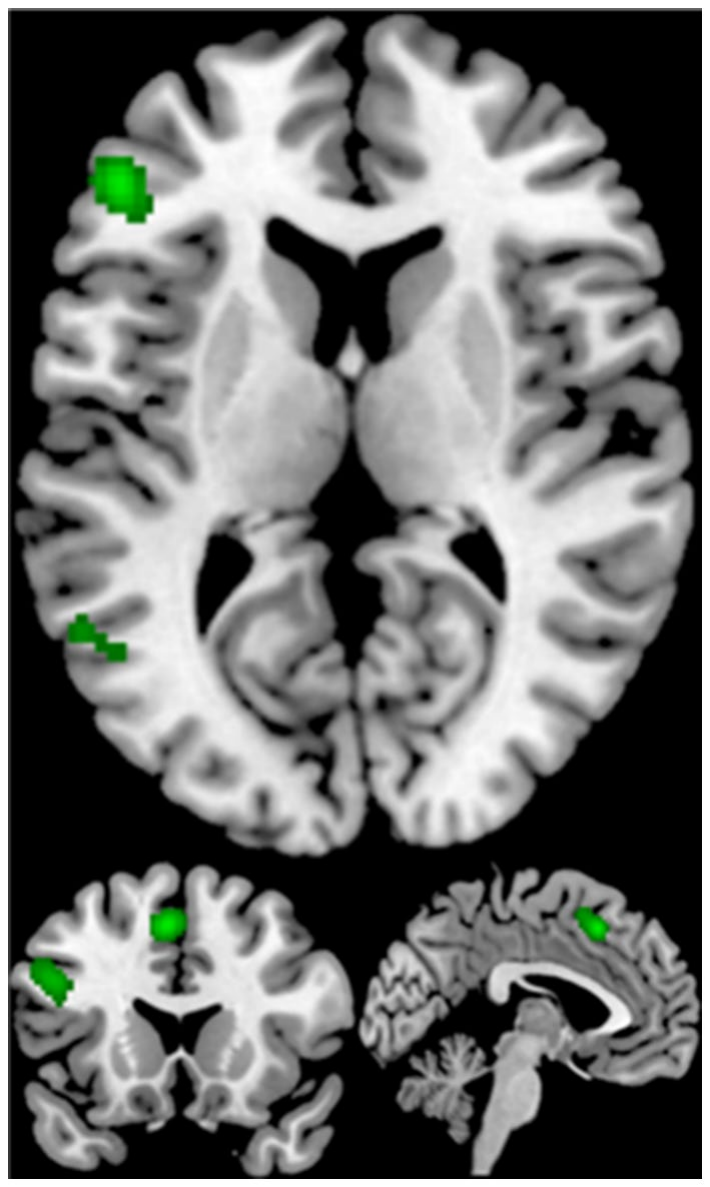
Displayed are significant results from the meta-analysis of creativity.

The ALE meta-analysis on 56 studies in creativity resulted in 4 significant clusters of activation, including the left inferior frontal gyrus, the left middle frontal gyrus, the left superior frontal gyrus, the left middle occipital gyrus, the left middle temporal gyrus, the left inferior parietal lobule (see Table 4; Figure 3).

**Table 4:** Regions of activations resulting from the meta-analysis of creativity.

Cluster	Vol (mm^3^)	Region	H*	BA*	MNI coordinates			ALE
					x	y	z	
1	6,456	Inferior Frontal Gyrus	L	9	−46	12	30	0.0453
		Middle Frontal Gyrus	L	46	−48	38	12	0.0413
		Middle Frontal Gyrus	L	46	−42	28	18	0.0303
		Inferior Frontal Gyrus	L	9	−44	4	20	0.0292
		Middle Frontal Gyrus	L	9	−52	22	30	0.0290
2	1,552	Superior Frontal Gyrus	L	6	−4	18	48	0.0410
3	1,480	Middle Occipital Gyrus	L	19	−50	−60	−8	0.0290
		Middle Temporal Gyrus	L	*	−50	−66	2	0.0280
		Middle Temporal Gyrus	L	39	−56	−56	10	0.0248
		Middle Temporal Gyrus	L	37	−50	−60	8	0.0221
4	1,352	Inferior Parietal Lobule	L	40	−58	−30	40	0.0426

### 3.2 ALE meta-analysis

**Figure 4:**
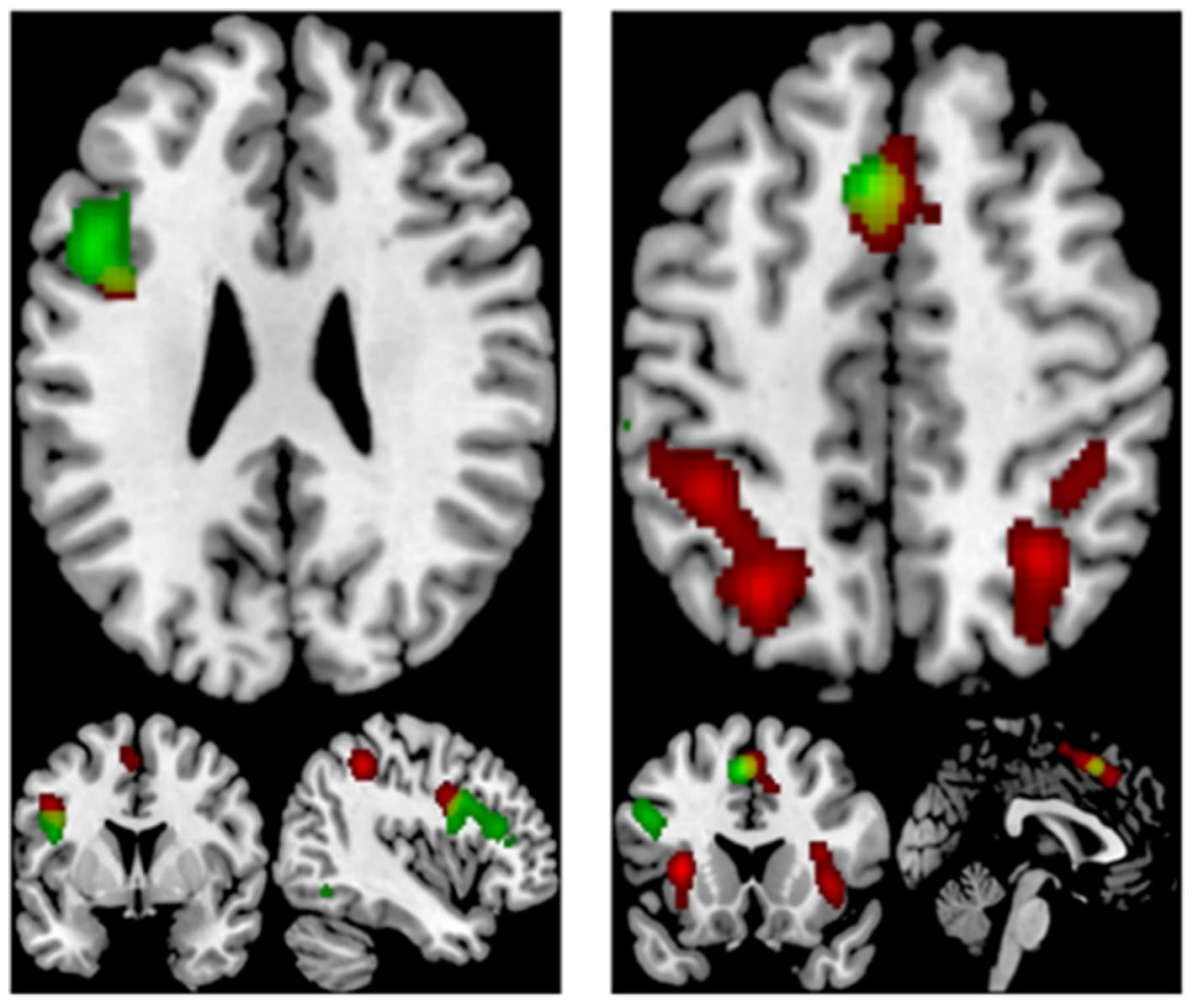
Displayed are significant results from the meta-analysis common of mathematics and creativity.

The contrast analysis for increased activation during mathematics compared to creativity with a total of 5 clusters, including the bilateral precuneus, the bilateral claustrum, the right superior parietal lobule, the right angular gyrus, the right insula, and the right inferior parietal lobule. Creativity revealed 3 clusters of significant activation compared to mathematics. The left inferior frontal gyrus, the left inferior parietal lobule, and the left middle frontal gyrus are included. The common brain regions activated by both mathematical and creative tasks were the left inferior frontal gyrus and the left superior frontal gyrus (see Table 5; Figure 4).

**Table 5:** Regions of activations resulting from the contrast analysis of the conditions “mathematics” and “creativity.”

Cluster	Vol (mm^3^)	Region	H*	BA*	MNI coordinates			ALE
					x	y	z	
Mathematics > Creativity								
1	2,128	Precuneus	R	7	28	−61.5	45	3.7190
		Precuneus	R	7	28	−58	38	3.7190
		Precuneus	R	7	28	−70	46	3.4316
		Angular Gyrus	R	39	34	−56	36	3.3527
		Precuneus	R	7	28	−61	33	2.9888
		Superior Parietal Lobule	R		30	−72	54	2.3697
2	1832	Claustrum	R		34	16	−4	3.8905
		Insula	R	13	32	30	6	3.4316
		Claustrum	R		34	16	2	3.2388
		Insula	R	13	35	22	3	2.7821
3	1,504	Precuneus	L	7	−25.7	−60.8	41.9	3.8905
4	1,464	Claustrum	L		−29.6	18.4	3.8	3.8905
5	272	Superior Parietal Lobule	R	7	34	−48	42	2.6874
		Inferior Parietal Lobule	R	40	38	−48	42	2.5758
		Inferior Parietal Lobule	R	40	38	−48	48	2.3534
Mathematics < Creativity								
1	976	Inferior Frontal Gyrus	L	9	−49.5	10.7	20.4	3.8905
2	976	Inferior Parietal Lobule	L	40	−59.6	−29.3	40.2	3.3527
3	800	Middle Frontal Gyrus	L	46	−42.7	33.3	12	3.8905
		Inferior Frontal Gyrus	L	46	−52	34	12	3.1946

### 3.3 MACM analysis

**Figure 5:**
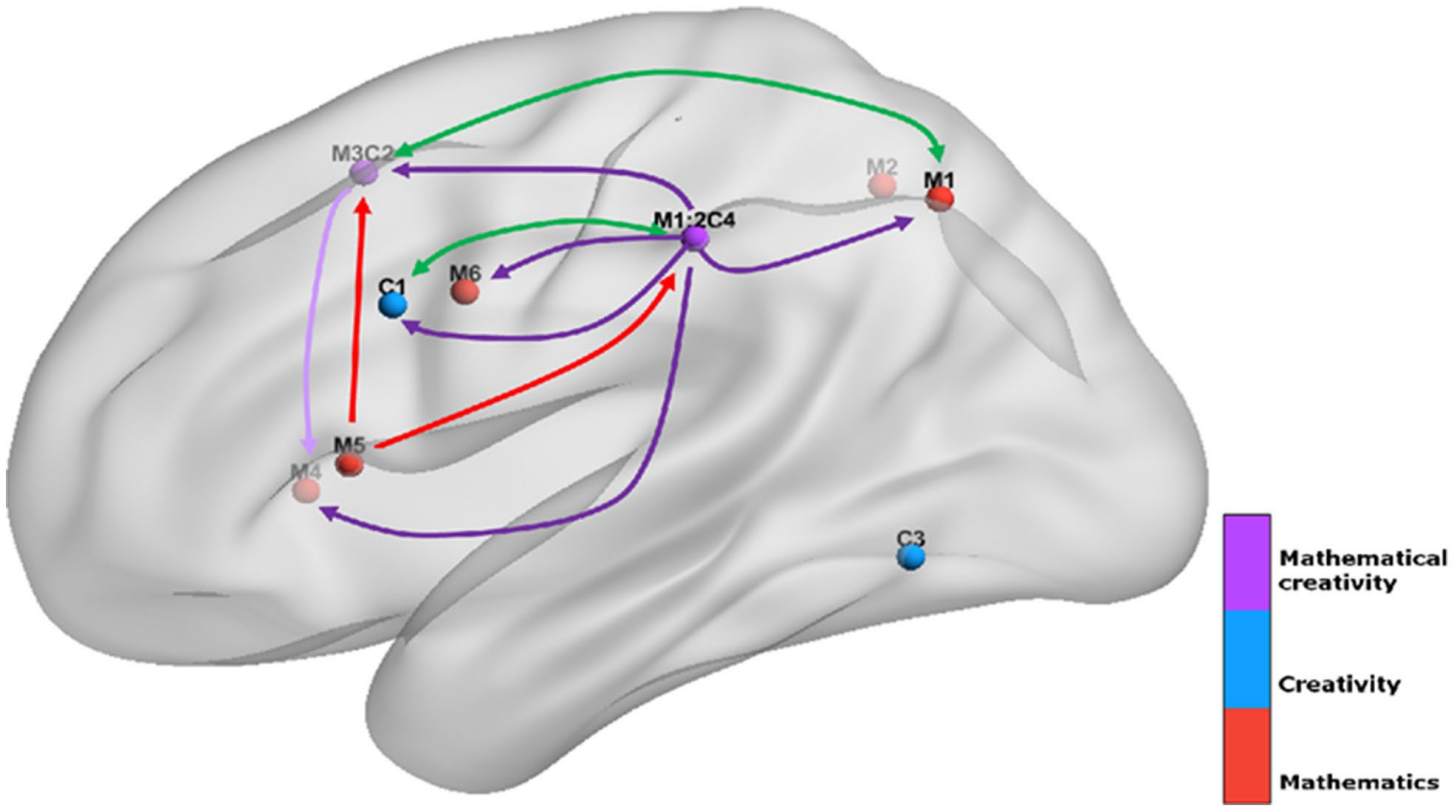
Co-activation network among 9 ROIs related to mathematical creativity region.

The focus of this study is on the cognitive neural mechanisms of mathematical creativity, so the results of the MACM analyses of the left inferior parietal lobule (IPL) and the left superior frontal gyrus (SFG), the regions where mathematical ability and creativity are co-activated, will be analyzed specifically. Results related to other regions can be viewed in the Appendix. Co-activation maps for the left inferior parietal lobule were significant for the right inferior parietal lobule (BA 40), the bilateral medial frontal gyrus (BA 6), the right sub-gyral (BA 6), the left middle frontal gyrus (BA 6), the left insula (BA 13, 47), the right claustrum, the left inferior frontal (BA 9), the right superior temporal gyrus (BA 41, 22) (see Table 5; Figure 5). For the left superior frontal gyrus significant co-activation was observed in the right angular gyrus (BA 39), the left middle frontal gyrus (BA 9), the left precuneus (BA 19), the left inferior parietal lobule (BA 40), the left sub-gyrus (BA 6), the bilateral middle frontal gyrus (BA 6), and the left insula (BA 13) (see Tables 6, 7; Figure 5).

**Table 6:** MACA results: regions of functional coactivation associated with left Inferior Parietal Lobule.

Cluster	Vol (mm^3^)	Region	H*	BA*	MNI coordinates			ALE
					x	y	z	
1	12,584	Inferior Parietal Lobule	L	40	−40	−44	44	0.0809
		Inferior Parietal Lobule	L	40	−48	−52	48	0.0417
		Precuneus	L	7	−26	−64	46	0.0372
		Postcentral Gyrus‑	L	3	−36	−26	52	0.0231
2	4,696	Inferior Parietal Lobule	R	40	36	−52	46	0.0494
		Inferior Parietal Lobule	R	40	48	−38	48	0.0244
3	3,744	Medial Frontal Gyrus	L	6	−4	12	54	0.0548
		Medial Frontal Gyrus	L	6	−2	0	58	0.0221
		Medial Frontal Gyrus	R	6	8	12	50	0.0199
4	2,960	Sub-Gyral	R	6	30	2	56	0.0397
		Precentral Gyrus	R	6	40	−8	48	0.0295
5	2,848	Middle Frontal Gyrus	L	6	−26	−4	52	0.0332
		Sub-Gyral	L	6	−28	4	56	0.0319
		Sub-Gyral	L	6	−16	0	56	0.0241
		Middle Frontal Gyrus	L	6	−22	4	64	0.0227
6	2,672	Insula	L	13	−32	20	4	0.0619
		Insula	L	47	−30	22	−10	0.0252
7	2,544	Claustrum	R	*	34	20	4	0.0543
8	2,512	Inferior Frontal Gyrus	L	9	−50	8	20	0.0291
		Middle Frontal Gyrus	L	9	−52	10	36	0.0286
		Precentral Gyrus	L	6	−44	4	30	0.0233
		Inferior Frontal Gyrus	L	9	−54	12	26	0.0229
9	1,208	Superior Temporal Gyrus	R	41	62	−22	4	0.0289
	1,208	Superior Temporal Gyrus	R	22	56	−12	8	0.0226

**Table 7:** MACA results: regions of functional coactivation associated with left Superior Frontal Gyrus.

Cluster	Vol (mm^3^)	Region	H*	BA*	MNI coordinates			ALE
					x	y	z	
1	3,312	Superior Frontal Gyrus	L	6	−4	14	52	0.0579
		Superior Frontal Gyrus	L	6	−6	26	62	0.0163
2	1,480	Angular Gyrus	R	39	34	−56	44	0.0292
3	1,432	Middle Frontal Gyrus	L	9	−42	26	30	0.0269
		Inferior Frontal Gyrus	L	9	−42	10	26	0.0227
4	1,192	Precuneus	L	19	−28	−70	42	0.0308
5	1,152	Inferior Parietal Lobule	L	40	−36	−48	44	0.0272
6	1,144	Sub-Gyral	L	6	−28	4	56	0.0241
		Middle Frontal Gyrus	L	6	−28	−2	62	0.0189
		Middle Frontal Gyrus	L	6	−22	4	66	0.0173
7	1,096	Middle Frontal Gyrus	R	6	32	4	60	0.0273
8	1,040	Insula	L	13	−34	20	2	0.0269

## 4 Discussion

### 4.1 ALE meta-analysis

The results of the ALE for creative thinking showed that the activated brain regions were mainly concentrated in the left hemisphere, which included the inferior frontal gyrus (BA 9, 46), the superior frontal gyrus (BA 6), the middle occipital gyrus (BA 19), and the inferior parietal lobule (BA 40) regions. It has been shown that the IFG generates creative ideas through the retrieval of loosely related semantic concepts, the screening of creative ideas, and the evaluation of originality (Cogdell-Brooke et al., 2020). Numerous previous meta-analyses of creative thinking have also found this region to be activated because it involves integrating semantic concepts and ideas in new ways (Boccia et al., 2015; Wu et al., 2015). The SFG has also been shown to be activated in numerous meta-analyses, and existing meta-analyses suggest that this region may be activated in different creative tasks because of its need to flexibly manipulate semantic information, so that the generation of many original ideas is associated with this region (Cogdell-Brooke et al., 2020). The SFG plays an important role in cognitive functions such as working memory, mental manipulation, and spatial orientation processing, and there is a stable correlation between this region and creative thinking skills, especially with insight (Lin et al., 2018). The MOG is primarily associated with mental imagery, which plays an important role in creative thinking (Beaty et al., 2018). The IPL is an important region for semantic processing and has been shown to play a key role in situational memory retrieval, and cognitive operations that facilitate situational retrieval can be effective in promoting creative thinking (Madore et al., 2016). Also, this region has been shown to promote divergent thinking (Kuang et al., 2022). These activation regions suggest that prefrontal regions are critical in creativity. And the importance of this region has been repeatedly demonstrated in individuals with prefrontal damage, where creativity is also impaired to some extent (Khalil and Moustafa, 2022; Ovando-Tellez et al., 2019).

The results of the ALE for mathematics cognition revealed that the activated brain regions were predominantly located in the bilateral inferior parietal lobule (IPL) (BA 40), the left superior parietal lobule (SPL) (BA 32), the left superior frontal gyrus (SFG) (BA 6), the right medial frontal gyrus (MFG) (BA 8), the left medial frontal gyrus (MFG) (BA 6), the bilateral insula, and the left precentral gyrus. The meta-analysis of mathematical brain region associations in the present study indicated that the frontal–parietal region plays a key role in mathematical ability, which is generally consistent with the results of the prior meta-analysis (Istomina and Arsalidou, 2024). The IPL, particularly in its bilateral manifestation, is crucial for numerical processing and arithmetic reasoning. This region is known to be involved in the manipulation and representation of numerical quantities, as well as in spatial attention and number sense (Hubbard et al., 2005). The SPL, especially on the left side, plays a significant role in visuospatial processing and the integration of sensory information, which are essential in understanding and solving mathematical problems (Friedman and Miyake, 2017). The SFG, located in the left hemisphere, is associated with higher cognitive functions such as working memory and executive control, both of which are integral to complex mathematical problem-solving and abstract reasoning (Christoff et al., 2009). The MFG, particularly in the right hemisphere, contributes to decision-making and cognitive control, which are vital in tasks requiring logical reasoning and the application of mathematical principles (Arsalidou and Taylor, 2011). On the contrary, the left MFG is implicated in processes related to calculation and mathematical operations, reflecting its role in more structured and rule-based aspects of mathematics. The bilateral insula is involved in risk and uncertainty assessment, which can be crucial in mathematical decision-making and problem-solving scenarios (Preuschoff et al., 2008). The left precentral gyrus plays a role in motor planning and execution, and its involvement in mathematical cognition might be related to the mental manipulation of numbers and symbols, a process often metaphorically described as ‘mental gymnastics’ (Andres et al., 2008). These activation regions emphasize the multifaceted nature of mathematical cognition, involving a network of brain areas responsible for numerical processing, spatial reasoning, working memory, decision-making, and symbolic manipulation. This complex interplay of cognitive functions highlights the integral role of these regions in the understanding and application of mathematics.

The results of the ALE for both mathematics and creativity indicated that the activated brain regions were crucially located in the left inferior frontal gyrus (IFG) and the superior frontal gyrus (SFG). The IFG plays a key role in generating creative mathematical ideas by retrieving and integrating diverse mathematical concepts, thereby fostering innovative problem-solving approaches (Beaty et al., 2016). Its ability to manipulate and recombine existing mathematical knowledge is essential for producing original and effective solutions. Meanwhile, the SFG is vital for higher cognitive functions like working memory and executive control, which are integral to mathematics and creativity. It helps maintain and manipulate complex mathematical information, enabling the exploration of various problem-solving strategies and their evaluation (Dietrich and Kanso, 2010). The SFG is particularly important in abstract mathematical reasoning and in shifting between different problem-solving methods. In addition, the SFG’s role in attentional control is important in maintaining focus on complex mathematical tasks, thus facilitating deeper engagement and persistence in finding creative solutions (Zabelina and Robinson, 2010). The combined functions of the IFG and SFG highlight the intricate cognitive processes involved in both the generation of novel ideas and the sustained manipulation and evaluation of these ideas. These activated brain regions associated with creativity and math are largely similar to previous meta-analysis results, but this study not only explored the brain regions, but also further explored the brain networks associated with math creativity through the MACM method.

### 4.2 MACM analysis

The results of the MACM analysis indicate that the frontoparietal network has a key role in mathematical creativity. This region is a core network for cognitive control and plays an important role in the processes of goal orientation, working memory, inhibitory switching, attention, cognitive control, and other cognitive abilities (Uddin et al., 2019). It has been shown that the frontoparietal network is the core network for creative performance and that stimulation of the frontoparietal network can effectively promote the enhancement of an individual’s creative ability (Lifshitz-Ben-Basat and Mashal, 2021; Tabatabaeian and Jennings, 2023). In addition, greater activation of this region also occurred when individuals solved math problems. Intervention training for children with math deficits has also shown that increased activation in the frontal–parietal region was found in these children after two weeks of numeracy training (Liang, 2022; Soltanlou et al., 2022). Recent research suggests that functional connectivity within the frontoparietal network plays a key role in mathematical ability and that individuals with high levels of mathematical ability may rely on this network for effective neural communication and information processing (Ren and Libertus, 2023). It can be seen that the frontoparietal network not only plays an important role in mathematics and creativity skills but also has a role in mathematical creativity that cannot be ignored.

## 5 Conclusion

Findings from the current meta-analysis on mathematical creativity provide valuable insights into the neural mechanisms underlying this complex cognitive process. Specifically, we focused on exploring brain regions associated with mathematical creativity using the ALE and MACM analytic approaches. Based on the ALE meta-analysis, we identified key brain regions activated during mathematical and creative tasks, with a particular focus on the left IFG and SFG. These findings were further substantiated by an MACM analysis, which highlighted the frontoparietal network’s critical role in mathematical creativity. These results demonstrate that the left IFG and SFG are essential in both mathematical reasoning and creative thinking. The IFG facilitates the generation of innovative mathematical ideas through the integration of diverse concepts, while the SFG supports higher cognitive functions such as working memory and executive control, essential for complex problem-solving in mathematics. The overlap in these regions during tasks requiring mathematical and creative cognition underscores their importance in the cognitive processes that underlie mathematical creativity. Compared to creativity tasks, mathematical tasks show increased concordance in the precuneus, claustrum, and superior parietal lobule. On the other hand, creativity tasks show increased concordance in the inferior frontal gyrus, inferior parietal lobule, and middle frontal gyrus. Moreover, the frontoparietal network, identified as a core network for cognitive control, was found to be significantly activated in tasks involving mathematical creativity. This network’s involvement in goal orientation, working memory, and cognitive control suggests its integral role in both domain-specific mathematical abilities and general creative performance.

To foster mathematical creativity, teachers can be suggested to use students’ episodic knowledge about the problem-solving process, an abacus, diagrams, gestures, graphs, symbols, etc., as visualization while explaining mathematical contents, and speech with gesture strategy when students solve the problems. In addition, not only focused on cognitive aspects, teachers help students build intrinsic motivation and affective goals to cause their effort in attention and complex processing. Our study contributes to the ongoing efforts to understand and enhance creativity in educational settings, particularly within the domain of mathematics. Our findings may shed light on potential avenues for the development of effective educational strategies to promote students’ mathematical creativity.

## Data Availability

The original contributions presented in the study are included in the article/supplementary material, further inquiries can be directed to the corresponding author.

## Footnotes

^1^ http://www.brainmap.org/

^2^ http://ric.uthscsa.edu/mango/

^3^ https://www.nitrc.org/projects/bnv/
