# Corrigendum: Meta-analysis of fMRI studies related to mathematical creativity

**DOI:** 10.3389/fpsyg.2025.1580954

**Published:** 2025-03-11

**Authors:** Qingqing Li, Sungyeun Kim

**Affiliations:** ^1^Department of Psychology, Northwest Normal University, Lanzhou, Gansu, China; ^2^Graduate School of Education, Incheon National University, Incheon, Republic of Korea

**Keywords:** creativity, fMRI, mathematics, mathematical creativity, meta-analysis

In the published article, there was an error in affiliation 2. Instead of “Department of Education, Incheon National University, Incheon, Republic of Korea”, it should be “Graduate School of Education, Incheon National University, Incheon, Republic of Korea”.

The authors apologize for this error and state that this does not change the scientific conclusions of the article in any way. The original article has been updated.

